# Dynamic Ocular Surface and Lacrimal Gland Changes Induced in Experimental Murine Dry Eye

**DOI:** 10.1371/journal.pone.0115333

**Published:** 2015-01-15

**Authors:** Bing Xiao, Yu Wang, Peter S. Reinach, Yueping Ren, Jinyang Li, Shanshan Hua, Huihui Lu, Wei Chen

**Affiliations:** 1 School of Ophthalmology and Optometry, Wenzhou Medical University, Zhejiang, China; 2 Department of Biological Sciences, College of Optometry, State University of New York, New York, NY 10036, United States of America; National Institute of Dental and Craniofacial Research, UNITED STATES

## Abstract

Dry eye disease can be a consequence of lacrimal gland insufficiency in Sjögren’s Syndrome or increased tear film evaporation despite normal lacrimal gland function. To determine if there is a correlation between severity effects in these models and underlying pathophysiological responses, we compared the time dependent changes in each of these parameters that occur during a 6 week period. Dry eye was induced in 6-week-old female C57BL/6 mice by exposing them to an Intelligently Controlled Environmental System (ICES). Sixty mice were housed in ICES for 1, 2, 4 and 6 weeks respectively. Twelve were raised in normal environment and received subcutaneous injections of scopolamine hydrobromide (SCOP) 3 times daily for 5 days. Another sixty mice were housed in a normal environment and received no treatment. Corneal fluorescein staining along with corneal MMP-9 and caspase-3 level measurements were performed in parallel with the TUNEL assay. Interleukin-17(IL-17), IL-23, IL-6, IL-1, TNF-α, IFN-γ and TGF-β2 levels were estimated by real-time PCR measurements of conjunctival and lacrimal gland samples (LGs). Immunohistochemistry of excised LGs along with flow cytometry in cervical lymph nodes evaluated immune cell infiltration. Light and transmission electron microscopy studies evaluated LGs cytoarchitectural changes. ICES induced corneal epithelial destruction and apoptosis peaked at 2 weeks and kept stable in the following 4 weeks. In the ICES group, lacrimal gland proinflammatory cytokine level increases were much lower than those in the SCOP group. In accord with the lower proinflammatory cytokine levels, in the ICES group, lacrimal gland cytosolic vesicular density and size exceeded that in the SCOP group. ICES and SCOP induced murine dry eye effects became progressively more severe over a two week period. Subsequently, the disease process stabilized for the next four weeks. ICES induced local effects in the ocular surface, but failed to elicit lacrimal gland inflammation and cytoarchitectural changes, which accounts for less dry eye severity in the ICES model than that in the SCOP model.

## Introduction

Dry eye (DE) disease therapeutic management can be limited to providing palliative relief since its underlying mechanisms are not fully understood [1–3]. Topical ophthalmic cyclosporine can be an effective treatment as it targets immunopathological mechanisms. In recent years, the identification of an immune component to this disease sparked efforts to delineate how infiltrating immune cells give rise to this condition [4]. There are two major types of dry eye: aqueous-deficient dry eye (ADDE) and evaporative dry eye (EDE) models used for this purpose [5]. ADDE is characterized by a lack of tear production and secretion by the lacrimal glands [6], while EDE is caused by excessive tear evaporation, which leads to tear film instability with normal tear production. Conditions that underlie the development of the EDE model are becoming more prevalent in the human environment. They include increased exposure to environmental stresses such as excessive air conditioner mediated temperature lowering and emerging dependence on visual display terminal (VDT) usage for work and recreation [7]. In addition, more individuals are seeking symptomatic relief from this syndrome because they are becoming more aware of the potential hazards to ocular health maintenance by leaving this disease untreated.

The EDE model mimics anterior ocular surface dryness caused by excessive tear film evaporation resulting from housing animals in a stable and constant low humidity environment having high airflow and constant ambient temperature of about 22°C, whereas scopolamine hydrobromide (SCOP) model is a tear deficient model that mimics declines in lacrimal gland secretory activity resulting from immune cell infiltration in Sjögren’s Syndrome. The latter model is established by repeated injections of scopolamine, which by blocking acetylcholine-induced parasympathetic lacrimal gland secretory activity elicits functional and pathologic changes in the ocular surface [8–11]. The pathogenic events underlying the immune components in these two disease models leading to chronic inflammation are unique for each of the two models. Unraveling the immune cell response dynamics provides promise for identifying potential novel drug targets to better control this disease.

Dry eye (DE) is frequently characterized by variable amounts of ocular surface inflammation in the SCOP animal model [12]. This response is associated with enhanced proinflammatory cytokine expression (e.g., IL-1, IL-6, IL-8, TNF-alpha) along with compromise of ocular surface epithelial integrity and tear film secretion and content deficiencies [13, 14]. Even though there is widespread and large inflammatory cell infiltration in the lacrimal gland in Sjögren’s patients, there are no reports describing inflammation infiltration in the EDE model [15–17].

We compare here in the SCOP and EDE dry eye disease mouse models the time dependent changes for up to 6 weeks in proinflammatory IL, TNF-α and IFNγ and anti-inflammatory TGFβ-2 conjunctival gene expression. Along with profiling these changes, we evaluated the associated effects of these model-induced stresses on corneal epithelial barrier function as well as integrity, apoptotic activity and lacrimal gland cytoarchitecture. Even though in the EDE model the increases in lacrimal gland proinflammatory gene expression were less than those in conjunctival tissues of the SCOP model, in the lacrimal glands secretory vesicle retention was more evident in the EDE than the SCOP model. Taken together, proinflammatory increases in gene expression of Th1- and Th17-associated cytokines underlie much of the immunological responses in these two different models of dry eye disease. Stabilization of the increases in proinflammatory cytokine expression after two weeks suggests that concomitant rises in antiinflammatory lymphocytes counter any further increases from occurring during the subsequent month of study.

## Methods

### Animals

All procedures were approved by the Animal Care and Ethics Committee of Wenzhou Medical College, Zhejiang, China. The animals were humanely killed with an overdose of a mixture of ketamine and xylazine. All procedures were performed in accordance with the Association of Research and Vision in Ophthalmology (ARVO) statement for the Use of Animals in Ophthalmic and Vision Research. A total of 132 female C57BL/6 mice (age range, 4–6 weeks) were supplied by the Animal Breeding Unit of Wenzhou Medical College.

### ICES-induced murine dry eye model

ICES was established to induce dry eye as previously described [8–10]. This system was characterized with humidity of 13.1±3.5%, airflow of 2.2±0.2 m/s, and temperature of 22±2°C. An alternating 12-hour light–dark cycle (8 AM to 8 PM) was employed. Water and food were made available ad libitum.

### Grouping

Sixty mice were housed in ICES for 1, 2, 4 and 6 weeks respectively, and served as part of the experimental group (E). Twelve were maintained in a normal laboratory environment and received 3 times daily subcutaneous injections of 0.1 mL of 5 mg/mL scopolamine hydrobromide for 5 days (SCOP, Sigma-Aldrich Corp., St Louis, MO) [18], and served as SCOP group (SCOP). Another sixty mice were also housed in the normal laboratory environment (room temperature of 23±2°C, relative humidity of 60%±10%) but received no treatment and were designated as the normal control group (N).

### Corneal fluorescein staining

Fluorescein staining was performed on each group by instilling 0.5 µl of 5% fluorescein solution into the inferior conjunctival sac using a micropipette. The stained area was assessed and graded using the 2007 Dry Eye Work Shop (DEWS) recommended grading system by a masked observer. The corneas were rated from 0 to 4 with the cornea surface divided into five regions (0 dot, Grade 0; 1–5 dots, Grade 1; 6–15 dots, Grade 2; 16–30 dots, Grade 3; and 30 dots, Grade 4). The total score from the five regions was recorded.

### Immunofluorescent staining

The eyes from each group were excised, embedded in optimal cutting temperature compound (OCT compound; VWR, Suwanee, GA), and flash frozen in liquid nitrogen. Sagittal 8-μm sections were cut with a cryostat (HM 550; Microm, Waldorf, Germany) and placed on glass slides that were stored at—80°C. Tissues were fixed with methanol at 4°C for 10 min. After fixation, they were permeabilized with PBS containing 0.1% Triton-X for 10 min. Then they were blocked with 20% normal goat serum in PBS for 45–60 min. Primary polyclonal rabbit antibodies against MMP-9 (1:100 dilution; Abcam, Cambridge, MA) and caspase-3 (1:100 dilution, Abcam, MA, USA) were applied and incubated for 12 h at 4°C. Secondary antibodies, Alexa-Fluor 594-conjugated goat anti-rabbit IgG (1:300; Invitrogen, Molecular Probes, Eugene, OR) were then applied and incubated in a dark chamber for 1 h, followed by counter-staining with 4′,6-diamidino-2-phenylindole (DAPI, 1:1000 dilution) for 30 min. MMP-9 expression was observed and photographed with laser scanning confocal microscopy (LSM 710; Zeiss with krypton-argon and He-Ne laser; Carl Zeiss Meditec, Sartrouville, Germany).

### TUNEL assay

DNA fragmentation detected by TUNEL assay was evaluated by laser scanning confocal microscopy using frozen corneal tissue sections. Mice eyes from each group were excised. Corneal section slides were fixed with 4% paraformaldehyde in PBS at room temperature for 10 minutes. After fixation, they were permeabilized with Triton-X (0.1% in PBS, Sigma, St Louis, USA) for 10 minutes and then 50 μl (5 μl Enzyme solution in 45 μl Label solution) TUNEL reaction mixture (In Site cell Death Detection Kit, Roche, Mannhein, Germany) was applied and incubated for 1 hour at 37°C in a humidified atmosphere. Counter staining with DAPI (1:1000 dilution) was followed for 30 minutes. Sections were covered with antifade mounting medium and sealed with a cover slip for microscopic observation.

### RNA isolation and real-time PCR

Total RNA from conjunctivas and lacrimal glands was extracted (RNeasy mini kit (50x), Qiagen, Crawley, U.K.) according to the manufacturer’s instructions. Samples within each group were pooled. The RNA concentration was measured based on its optical density at 260 nm and stored at −80°C before use. cDNA was synthesized from 1 μg of total RNA using random primer and Moloney Murine Leukemia Virus reverse transcriptase. Quantitative real-time polymerase chain reaction (qRT–PCR) analysis was employed using the Power SYBR Green PCR Master Mix (Applied Biosystems, Paisley, UK) and Applied Biosystems 7500 Real-Time PCR System (Applied Biosystems). The primers are provided in Table 1. Assays were performed in duplicate and repeated three times using different samples from different experiments. The RT-PCR results were analyzed using the comparative threshold cycle (CT) method and normalized with glyceraldehyde 3-phosphate dehydrogenase (GAPDH) as an endogenous reference.

**Table 1:** Primers used for quantitative RT-PCR.

**Gene**	**Accession no.**	**Forward(5′-3′)**	**Reverse(5′-3′)**
IL-1β	NM_008361	tgagctgaaagctctccacc	ctgatgtaccagttggggaa
IL-6	NM_031168	agataacaagaaagacaaagccagagtc	gcattggaaattggggtaggaag
IL-17	NM_010552	ctcaaccgttccacgtcaccct	ccagctttccctccgcatt
IL-23	NM_031252	gcaccagcgggacatatgaa	caagcagaactggctgttgtc
IFN-γ	NM_008337	atgaacgctacacactgcatc	taggctttcaatgactgtc
TNF-α	NM_000594	tctactgaacttcggggtgatcg	cgtgggctacaggcttgta
TGF-β2	NM_009367	ctcccctccgaaaatgcca	gttttgcaagcggaagaccc
GAPDH	NM_001289726	tgtccgtcgtggatctgac	cctgcttcaccaccttcttg

### Histological Analysis

Each entire lacrimal gland was fixed in 10% formalin. After dehydration, the specimens were embedded in paraffin, cross-sectioned, and stained with hematoxylin-eosin reagent and viewed under a microscope (Imager.Z1; Carl Zeiss Meditec, Oberkochen, Germany). To prevent experimental bias, all of the photographs were taken at random and assessed by two independent researchers in a blind manner using Photoshop CS4 (Adobe Systems Inc, Tokyo, Japan) and software ImageJ 1.46r (National Institute of Health).

### Transmission electron microscopy (TEM)

LG tissue was fixed with 2.5% glutaraldehyde in 0.1 M phosphate buffer (pH 7.4) for 1 hour. Samples were then post-fixed in 1% osmium tetroxide in 0.1 M phosphate buffer at 4°C for one hour. The LG was dehydrated in graded ethyl alcohol series and embedded in Epoc 812. An ultrathin section was cut using a RT-7000 (RMC,USA), stained with uranyl acetate and lead citrate, and then examined with transmission electron microscopy (H-7500;HITACHI, Japan).

### Immunohistochemistry

Lacrimal glands were surgically excised and immersed in 4% paraformaldehyde overnight at 4°C. The tissue blocks were washed, dehydrated, embedded in paraffin, cut to a thickness of 3 mm. The cells were counted that stained positively for CD4 (clone H129.9, 10μg/mL; BD Bioscience, San Diego, CA), CD8α (clone 53e6.7, 3.125μg/mL; BD Bioscience), CD11b (clone M1/70, 6.25 μg/mL; BD Bioscience),CD45 (clone 30-F11, 10 μg/mL; BD Bioscience), CD103 (clone 2E7, 10 μg/mL; Biolegend, San Diego, CA), paraffin sections were stained with the abovementioned primary antibodies and appropriate biotinylated secondary antibodies (all from BD Pharmingen, San Diego, CA) using a staining kit ( Vectastain Elite ABC kit; Vector, Burlingame, CA) and reagents (Nova-Red; Vector). Secondary antibody alone and appropriate anti-mouse isotype (BD Biosciences) controls were also performed. Two sections from each animal were examined and photographed with a microscope (Imager.Z1; Carl Zeiss Meditec, Oberkochen, Germany). Positively stained cells were counted in the stroma of the LG using image-analysis software (NIS Elements Software, version 3.0 BR; Nikon). Results were expressed as the number of positive cells per mm2.

### Isolation of Cervical Lymph Nodes

Superficial cervical lymph nodes (CLNs) from each group were surgically excised, compresssed between two sterile frosted glass slides, and made into a single-cell suspension. Cell populations were individually collected, centrifuged at 1000 rpm for 5 minutes, filtered, and resuspended. Single cells were processed for flow cytometry as described below.

### Flow Cytometry Analysis

Single-cell suspensions from cervical lymph nodes (CLN) were cell surface stained with PE-anti-CD4 (clone H129.19, BD Pharmigen), negative controls consisted of cells stained with PE-isotype antibody (clone R35–95, BD Pharmigen). FITC-anti-CD8 (clone 53–6.7,BD Pharmigen), negative controls consisted of cells stained with FITC-isotype antibody (clone R35–95, BD Pharmigen). Cells were then resuspended in fixation-permeabilization solution (Cytofix/Cytoperm; BD Pharmingen). A BD FACS Calibur was used for flow cytometry, and data were analyzed using BD Diva Software (BD Pharmigen). These experiments were repeated twice.

### Statistical Analysis

Statistical analyses were performed using SPSS 13.0 software. One-way ANOVA with Bonferroni correction was used for comparison among groups with Gaussian distributed values. The Mann-Whitney U test was used to compare non-normally distributed values between groups. p<0.05 was considered statistically significant.  

## Results

### ICES Induces Corneal Epithelial Disruption

**Figure 1:**
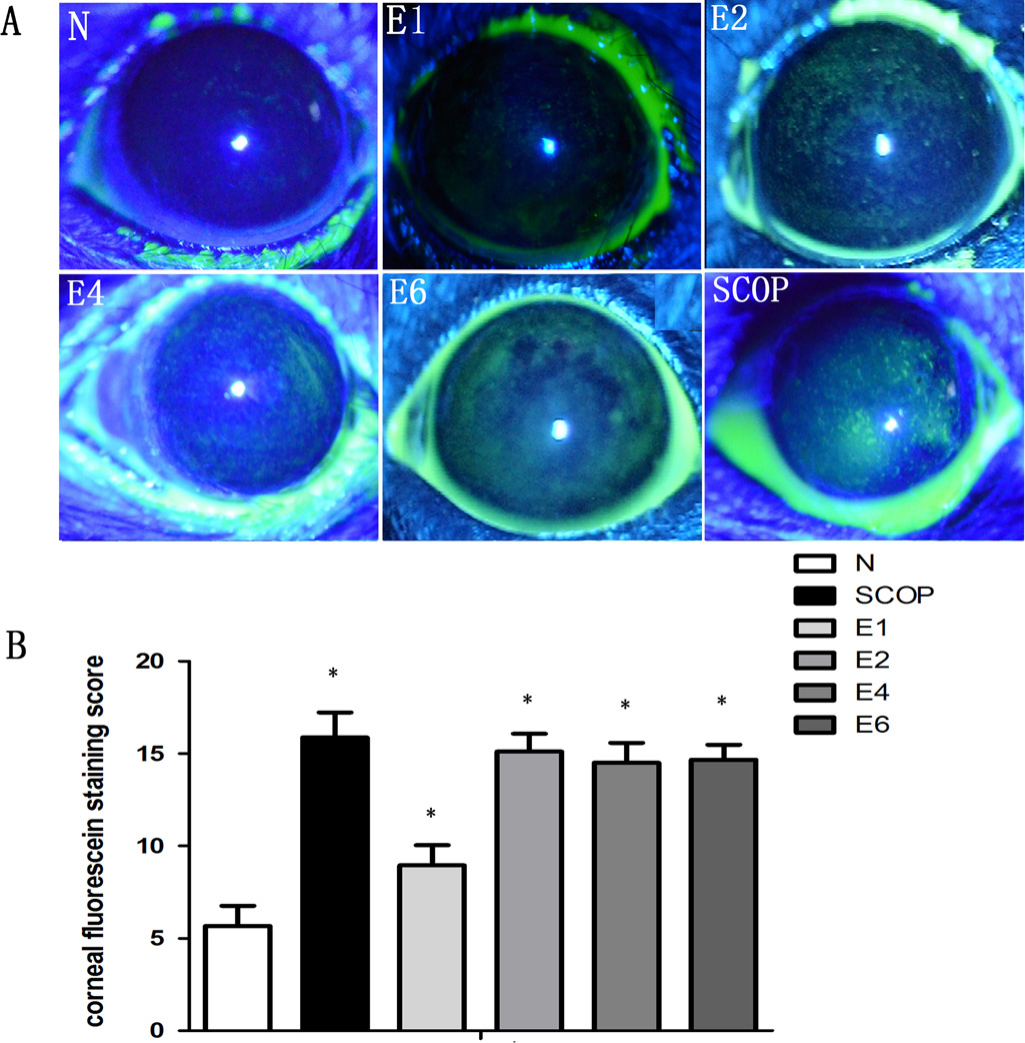
ICES Induced Corneal Epithelial Destruction. Corneal epithelial damage assessment by standard corneal fluorescein staining scores in ICES groups (E), the scopolamine-treated group (SCOP) and normal control group (N). *P < 0.05 versus the normal group (N).

**Figure 2:**
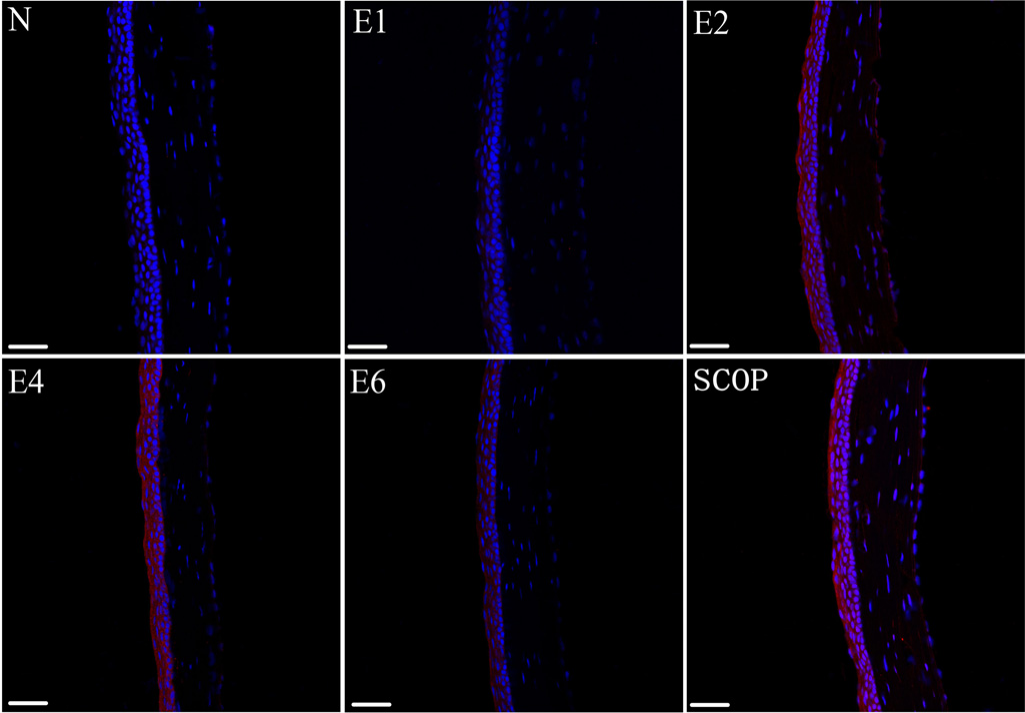
Matrix metalloproteinse 9 (MMP-9) expression profiles in each group. red, MMP9–positive; blue, DAPI-positive cells. (magnification:x20; scale bars: 50μm).

Fluorescein staining assessed changes in corneal epithelial integrity. In the ICES group, after 1 week there was a slight increase in the staining score which peaked at 2 weeks (P< 0.001) and was invariant for the next 4 weeks (Fig. 1). To evaluate if losses in the ICES group of tight junctional barrier function and epithelial integrity were accompanied by increases in MMP-9 expression, its staining pattern was also evaluated. In parallel with the development of corneal fluorescein staining, MMP-9 became the most intense after 2 weeks of ICES, which was unchanged during the following 4 and 6 weeks (Fig. 2). We also compared if there were differences in the development of MMP-9 expression in the ICES and SCOP groups. In the SCOP group, the MMP-9 expression was greater at all times than in the ICES group.

### ICES Induces Corneal Epithelium Apoptosis

**Figure 3:**
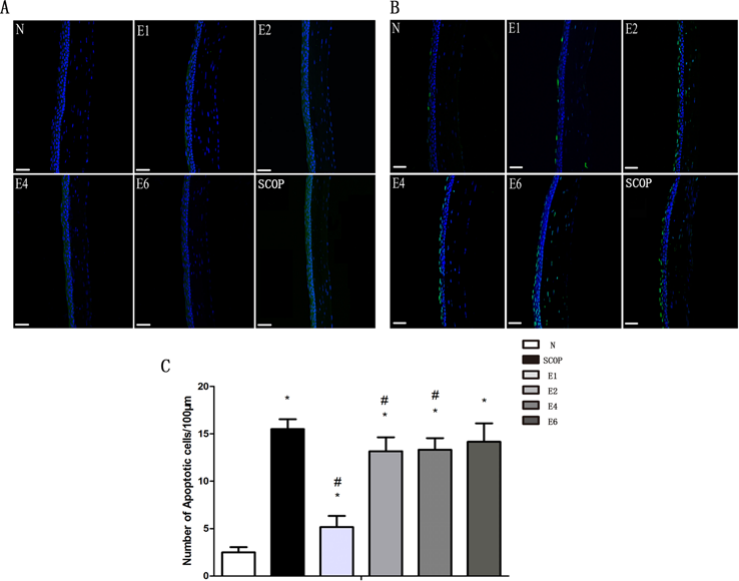
ICES Induced Corneal Epithelium Apoptosis. A,ICES induced caspase-3 expression in each group. (green, caspase-3–positive; blue, DAPI-positive cells) B, ICES induced TUNEL staining (green, positive to TUNEL staining). Magnification: x20; scale bars: 50μm. C, Graphs demonstrating the mean ± SD of apoptotic cell density in each group (number of cells / 100 μm). *P < 0.05 versus the normal group (N), #P < 0.05, versus the scopolamine-treated group (SCOP).

Caspase-3 immunofluorescence and TUNEL analyses were performed to evaluate the effect of ICES exposure on corneal epithelial apoptosis. Fig. 3A shows that caspase-3 expression increased after the first 2 weeks. It peaked already at 2-weeks and remained invariant at the 4-week and 6-week time points. Similarly, TUNEL results also demonstrated that the apoptotic cells increased in the ICES group, but remained unchanged at 2-, 4- and 6-weeks. (c.f. Fig. 3B, 3C). On the other hand, corneal epithelial apoptosis in the SCOP group seemed more pronounced at all of the same time points as those in the ICES group expect for a lack of a difference after 6 weeks.

### ICES Stimulates Inflammatory Cytokine Production in the Conjunctiva and Lacrimal Gland

**Figure 4:**
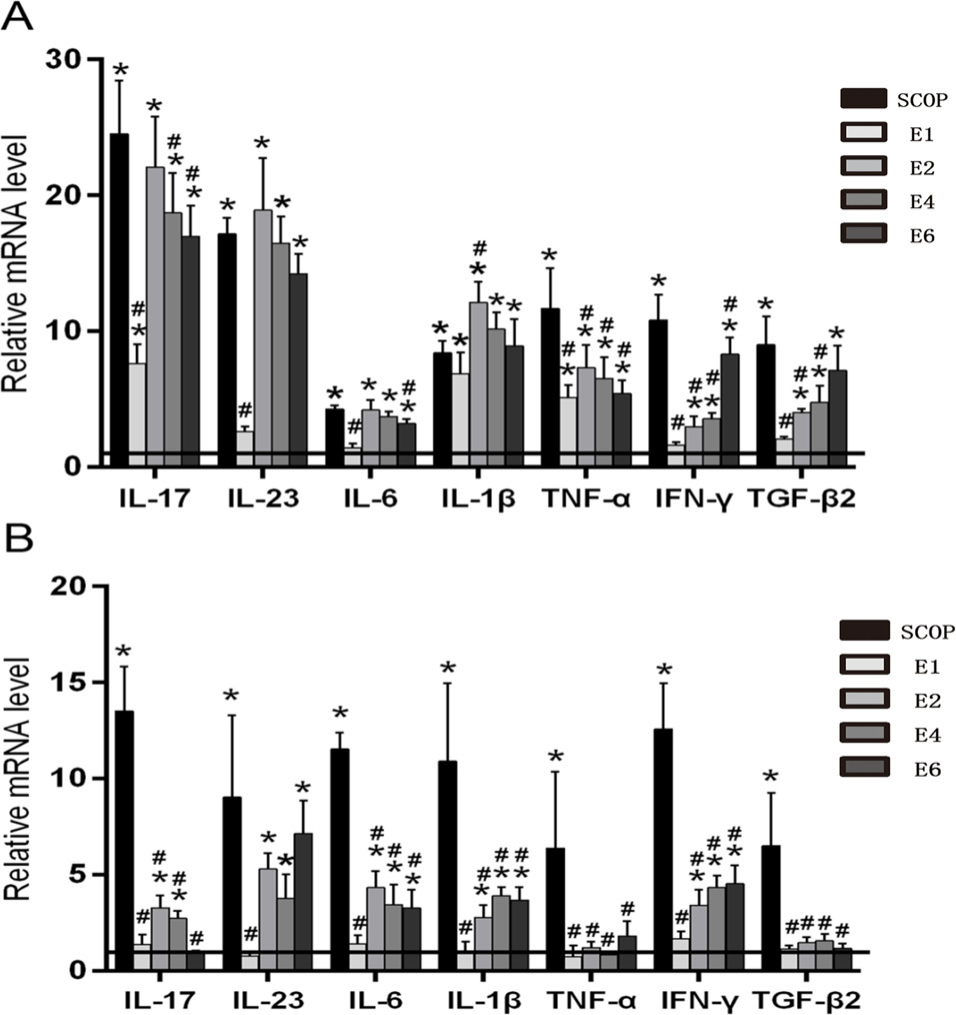
ICES Stimulates Inflammatory Cytokine Production in the Conjunctiva and Lacrimal Gland. A, Real-time PCR for the mRNA expression of IL-17, IL-23, IL-6, IL-1β, TNF-α, IFN-γ, TGF-β2 in the conjunctiva of normal group(Horizontal line), the scopolamine-treated group (SCOP), and after desiccating stress in ICES for 1 week,2week,4 week and 6week (E1,E2,E4,E6). B, mRNA transcript levels of IL-17,IL-23,IL-6,IL-1β, TNF-α, IFN-γ,TGF-β2 in the lacrimal gland. *P < 0.05 versus the normal group (N), #P < 0.05, versus the scopolamine-treated group (SCOP).

Levels of conjunctival IL-17, IL-23, IL-6, IL-1β, TNF-α mRNA transcripts peaked after 2 weeks in the ICES group without any further change during the subsequent 4 weeks. In contrast, conjunctival IFN-γ and TGF-β2 levels increased in the ICES group and peaked at 6-weeks. However, the gene transcript levels of all of these cytokines in the SCOP group were higher than those in the ICES group at all the time points (Fig. 4A).

The pattern changes in the transcript levels of all of these cytokines in the lacrimal gland of the ICES group are comparable to those in the conjunctiva. However, in the SCOP group at all the time points their levels were much higher compared to those in the ICES group (Fig. 4B).

### Lacrimal Gland inflammatory cell infiltration

**Figure 5:**
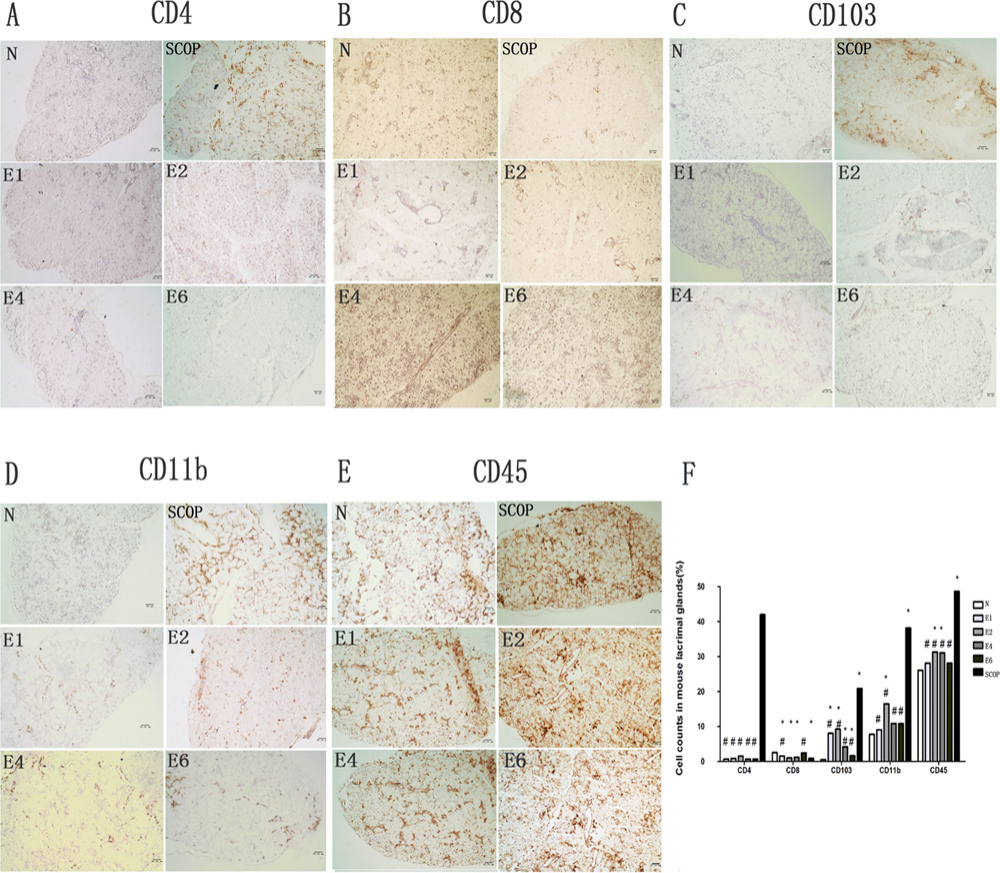
Inflammatory cells infiltration of the Lacrimal Gland. A-E, immunostained for CD4(A), CD8(B), CD103(C), CD11b(D), CD45(E) in the lacrimal glands sections. F, Cell counts in mouse lacrimal glands stained by immunohistochemistry for CD4, CD8, CD103, CD11b, CD45 sections in the normal group (N), the scopolamine-treated group (SCOP), and after desiccating stress in ICES for 1 week, 2 week, 4 week and 6 week (E1,E2,E4,E6). # P < 0.01 versus the scopolamine-treated group (SCOP), * P<0.05 versus the normal group(N) (Mann-Whitney U test). Original magnification:x20；scale bars = 50 μm. Experiments were repeated three times with two mice per group per experiment.

In order to further characterize differences in LG inflammation between the ICES and SCOP groups, we determined if increases in different inflammatory immune cell numbers correspond with rises in proinflammatory cytokine gene expression. Accordingly, we compared in the ICES and N groups CD4, CD8α immunohistochemistry (predominantly expressed on the surface of cytotoxic T cells), CD11b (a marker of monocyte/macrophage lineage), CD103 (a marker of intraepithelial lymphocytes) and CD45 (a marker of all leukocytes and largely naive T lymphocytes) positive T lymphocyte cell numbers (Fig. 5). CD8α cells in the ICES group were less than in the normal group (N). On the other hand, CD103 increased significantly after 1 week in the ICES group and remained elevated at the same level after 2-, 4- and 6-weeks, while CD4 levels in the ICES group remained at the baseline level at all the times. ICES group CD11b cells were the only ones that increased after 2 weeks. In the 2-week group, CD45 cell levels rose after 2 and 4-weeks. However, the numbers of infiltrated CD4, CD11b, CD103, CD45 cells in the SCOP group were much larger than those in the ICES group at all the times (Fig. 5).

### Lacrimal Gland structural changes induced by ICES

**Figure 6:**
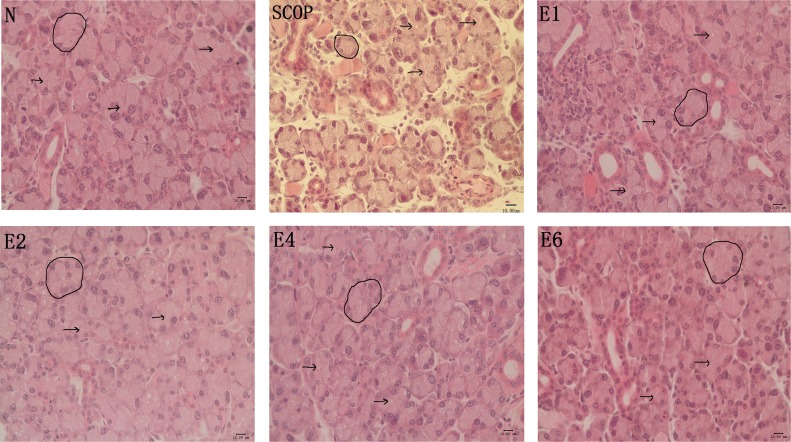
Lacrimal Gland Histology by H&E Staining. H&E staining of lacrimal gland from normal controls (N), the scopolamine-treated group(SCOP) and after desiccating stress in ICES for 1 week, 2week, 4 week and 6week (E1,E2,E4,E6). Arrows indicate ductal lumens. Circle indicates one acinus. Scale bars = 50μ m.

H&E staining showed that the acini of the lacrimal gland in the ICES group became slightly larger than those in the normal group after 2 weeks, without any further enlargement at either 4 or 6 weeks. But these changes were in sharp contrast with those occurring in the SCOP group. In the latter group, the acini were largely atrophic, replaced by fibrotic tissue and lymphocytic infiltrates (Fig. 6).

**Figure 7:**
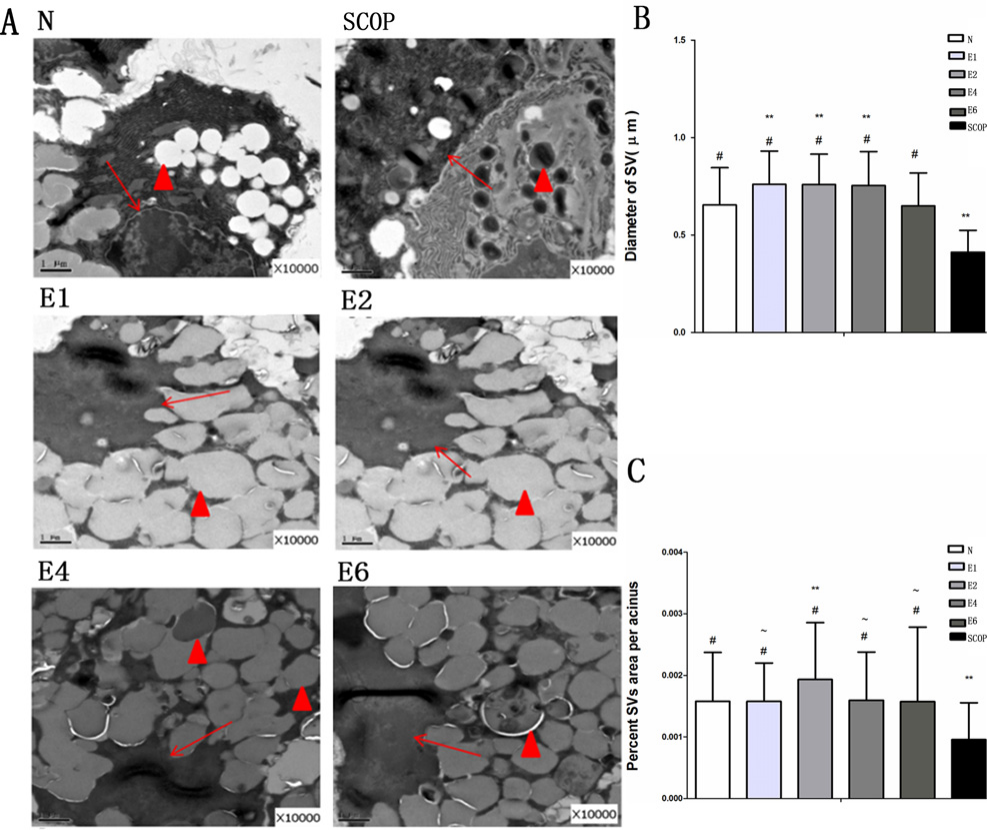
Electron microscopic findings of the lacrimal gland acinus. A, Secretory vesicle (SV) accumulation in the normal group(N), Scale bars = 1um. Atrophic SVs in the scopolamine-treated group (SCOP).Excessive accumulation of SVs in the 1-week in the E group (E1).Excessive accumulation of SVs in the 2-week group (E2). Excessive accumulation of SVs in the 4-week group (E4).Excessive accumulation of SVs in the 6-week group (E6). B: SV diameter. Total number of vesicles examined/group: 159/N group，254/SCOP group, 249/E1 group, 341/E2 group, 302/E4 group and 485/E6 group (Mann-Whitney U test). C: Percent SV area per acinus. Total number of acini examined/group: 148/N group ，177/SCOP group, 223/E1 group, 270/E2 group, 339/E4 group and 549/E6 group (Mann-Whitney U test) # P<0.001 versus the SCOP group, ** p<0.001 versus the normal group(N).~ P<0.001 versus the the E2 group. Original magnification: X10000. Hollow arrow, Ductal lumen; Arrows, Nuclei; Triangle, SV.

We then performed TEM to further resolve differences between lacrimal gland ultrastructural morphology in the ICES and N groups. In the ICES group at 1 week, there were more secretory vesicles (SVs) in the lacrimal gland epithelial cell cytoplasm, compared with the N group. They remained abundant in the ICES group at 2-, 4- and 6-weeks. On the contrary, in the SCOP group, they became atrophic (Fig. 7).

### ICES Does Not Cause Inflammation in Adjacent Lymph Nodes

**Figure 8:**
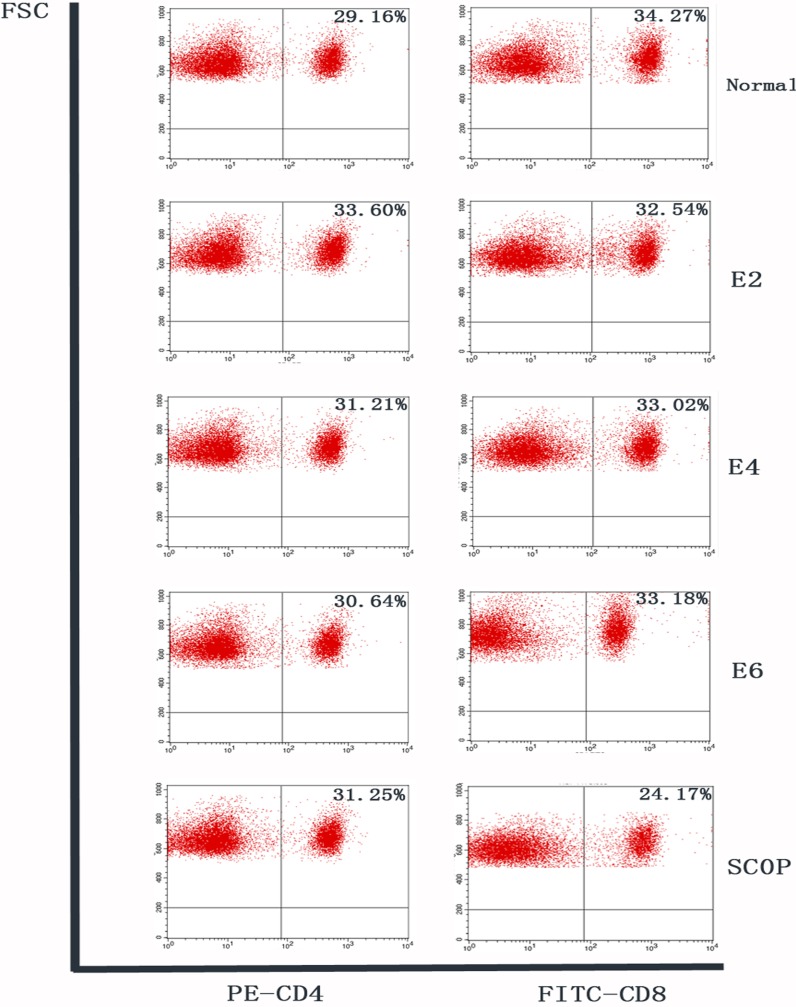
Flow cytometry analysis of cervical lymph nodes. Flow cytometry analysis of freshly isolated cells from cervical lymph nodes stained with CD4-PEand CD8-FITC conjugated antibody in each group. Numbers in the quadrants indicate the percentage of cells. Similar percentages of CD4+lymphocytes were observed in the cervical lymph nodes in all groups. Percentages of CD8+lymphocytes in ICES 2-week, 4-week and 6-week groups were also at baseline level, while that in the SCOP group was lower than the normal level.

Flow cytometry analysis of CLN cells stained for CD4 and CD8 was performed. Similar percentages of CD4+ lymphocytes were observed in these cells in the N, ICES and SCOP groups at all the different times. CD8+ lymphocyte percentages in the ICES group at 2-, 4- and 6-weeks were in all cases invariant at its baseline level, while in the SCOP group its level was even lower than in the N group (Fig. 8). 

## Discussion

This study was performed to further characterize and validate the usefulness of two different murine dry eye models of human DE disease. The ICES model is believed to model evaporative dry eye disease whereas the scopolamine model mimics Sjögren’s syndrome mediated lacrimal gland fibrosis and autoimmune rejection resulting in aqueous deficiency [8, 9, 17]. Our evaluation entailed contrasting and comparing the increases in proinflammatory cytokine gene expression, MMP-9 immunostaining, apoptosis, immune cell lacrimal gland infiltration as well as evaluation of the changes in lacrimal gland morphology at the light and electron microscopic levels that occur for up to 6 weeks after imposing either ICES or SCOP treatment conditions with effect. ICES elicited alterations in inflammation and apoptosis in the conjunctival epithelium, which mimic some of the changes occurring in human patients suffering from DE disease [19–22]. ICES also caused some changes in LGs structure and inflammation that were different from SCOP models. On the other hand, the SCOP model mimics in many ways the Sjögren’s syndrome condition in which the lacrimal gland undergoes immunorejection, atrophy as a consequence of larger increases in immune cell infiltration followed by rises in proinflammatory gene expression levels. This is associated with a more profound inflammatory response by the conjunctival epithelial cells along with losses in corneal epithelial integrity and rises in apoptosis. Our studies substantiate earlier indications that monitoring declines in ocular surface health induced by ICES for up to 2 weeks is sufficient to characterize DE disease development since during subsequent 4 weeks of observation DE indications almost stabilized. Nevertheless, our study provides a broader base for delineating the immunopathogenic changes resulting in the development of dry eye disease in two different relevant murine models. Our cataloging of the events underlying the plateauing of proinflammatory cytokine expression and immune cell infiltration between 2 and 6 weeks suggests that this stasis may be due to increases in anti-inflammatory cytokine expression which counterbalance the initial surge in proinflammatory cytokine expression.

Inflammation, corneal epithelial destruction and apoptosis can be induced in DE development [23–30]. We found that ICES induced losses in corneal epithelial integrity and apoptosis in a time dependent manner, which increased in the first 2 weeks and then remained invariant in the following 4 weeks. The peak level of ICES induced declines in corneal epithelial integrity and increases in apoptosis occurred at 2 weeks, which were comparable to those caused by scopolamine injection at 5 days.

Maintenance of healthy ocular immune microenvironment is dependent on a delicate balance between the factors eliciting proinflammatory and antiinflammatory events. This entails preventing proinflammatory lymphocytes (Th1 and Th17 types) from infiltrating into the eye to elicit increases in proinflammatory cytokine expression that overwhelms the ability of antiinflammatory lymphocytes (Th2 types and Tregs) to counter inflammation through rises in the release of suppressive interleukins (e.g.IL-4 and IL-10) and TGFβ-2 [31–40]. In accordance with the ocular surface symptoms, the transcriptional level of conjunctival pro-inflammatory cytokines including Th17 cell associated cytokine (IL-6, IL-23, and IL-17), IL-1β and TNFα rose and peaked at 2 weeks, which then remained invariant for up to 6 weeks. While the Th1 cell associated cytokine (IFN-γ) and the Treg (regulatory T cells) cell related cytokine (TGF-β2) displayed a different trend, which continuously increased up to 6 weeks. It is possible that the active Treg cell activation counteracted the elevated Th17 cell responses during the later 4 weeks, resulting in the 4-week plateau period of the ICES induced dry eye model. The immune suppressive functions of TGF-β-2 and Treg cells are extensively studied [41, 42]. Earlier studies found that TGF-β-2 could suppress T-cell proliferation by inhibiting the production of IL-2, a lymphokine known to potently activate T cells, NK cells, and other types of cells of the immune system [43]. Recently, TGF-β-2 was identified to be critical for the induction of IL-17 producing cells under inflammatory conditions [44–47]. Such evidence suggests that a functional balance between Tregs and effector T cells is vital to maintain efficient immune responses needed for preserving ocular surface health. We speculate that the plateau period from 2 weeks to 6 weeks of ICES was induced by the balanced status between Tregs and effector T cells.

De Paiva CS et al found significantly higher levels of IL-23 after 5 days of exposure to a desiccation stress. IL-6, IL-17 (both at 5 days and 10 days), IFN-γ (at 10 days) transcripts were higher in the conjunctiva of DE mice than the N group. TGF-β1 levels in conjunctival lysates increased significantly at 10 days, whereas TGF-β2 did not change [22]. In another study, higher levels of IL-17A, TGF-β1, TGF-β2, IL-6, IL-23, and IL-1 mRNA transcripts were observed in the corneal epithelium and conjunctiva of dry eye mice [48]. These results are consistent for the most part with ours except for somewhat larger increases in TGF-β2 levels in the aforementioned study. Pitcher et al proposed that elevated levels of IL-17A, IL-17R, IFN-γ, IL-6, IL-1β, and TNF-α transcripts were noted in SCOP2D mice and IFN-γ, TGF-β1, and IL-18R transcripts in SCOP5D mice. MMP-9, TGF-β2, did not change significantly in the SCOP model at any time point from 2 to 5 days [17].

In the lacrimal gland, the increases in proinflammatory cytokine gene expression levels exhibited similar trends to those occurring in the conjunctiva. However, the levels were significantly lower than those of the SCOP treated mice. Consistently, the CD4, CD11b, CD103 biomarker levels of infiltrating inflammatory cells including CD45 cells were also much higher in the SCOP group.

In the SCOP model, influx of CD4 T cells occurred into the parenchyma and periductal regions of the lacrimal gland, which is possibly associated with declines in acinar cell secretory activity. This pattern of changes is similar to that seen in SS patients. Such declines enhances exposure of lacrimal autoantigens to resident antigen presenting cells and initiates an autoimmune reaction. On the other hand, ICES induced local effects are restricted to the ocular surface, rather than mediating lacrimal gland inflammation and disruption of its cytoarchitecture. These differences may account for why pathology in the SCOP model are so much more severe than that in the ICES model. The SCOP model may be relevant to the condition in which cholinergic blockade induced by M3R autoantibodies in SS contributes to lacrimal gland inflammation. Because these autoantibodies appear capable of inhibiting cholinergic signaling as do anticholinergic agents such as scopolamine, it is possible that prolonged autoantibody-mediated cholinergic blockade could also promote lacrimal gland inflammation and secretory dysfunction.

Ultrastructural morphology analysis of the lacrimal gland showed that ICES caused increases in the number of secretory vesicles (SVs) in the cytoplasm of the epithelial cells, while those in the SCOP group were largely atrophic. Excessive accumulation of SVs, may be attributable to element and fluid entrapment. One possibility is that a decline in tear fluid secretion is essentially due to a decline in fluid secretion instead of fluid absorption into the gland. In contrast, the mechanism of SCOP—induced dry eye is due to both impaired tear production and secretion caused by impaired cholinergic support of lacrimal gland function.

Previous studies suggest that excessive SV accumulation in the lacrimal gland may contribute to the reduced tear secretion in some VDT users presenting with DE symptomology [49]. So it is possible that the ICES induced dry eye model, which mimics VDT dry eye patients, may cause tear secretion to decline due to suppression of SV content release.

Taken together, ICES induced murine dry eye develops from an initial surge in proinflammatory cytokine expression and immune cell infiltration that reaches a plateau after 2 weeks. It is sufficient to limit studies to this duration for the purpose of gaining additional insight into the pathogenic mechanisms that underlie DE disease development. Furthermore such an undertaking may lead to the identification of novel drug targets whose modulation will provide better control of the immune responses underlying this disease. On the other hand, to more clearly delineate the development of antiinflammatory mediator expression in these models, it may be more effective to extend the measuring period beyond two weeks. Such an extension may make it easier to better characterize their involvement in countering rises in proinflammatory cytokine expression and stabilizing DE disease progression.
